# Dimethyl­ammonium 5-carb­oxy-2-(1-oxo-1λ^5^-pyridin-2-yl)-1*H*-imidazole-4-car­box­yl­ate

**DOI:** 10.1107/S1600536812033557

**Published:** 2012-07-28

**Authors:** Chuntao Dai, Jianhua Nie, Yuehua Lin, Jun Wang

**Affiliations:** a Zhongshan Polytechnic, Zhongshan, Guangdong 528404, People’s Republic of China

## Abstract

In the title salt, C_2_H_8_N^+^·C_10_H_6_N_3_O_5_^−^, the imidazole­carboxyl­ate anion is essentially planar [maximum deviation from the least-squares plane = 0.046 (5) Å], with a dihedral angle between the rings of 2.7 (2)°. This conformation is maintained by the presence of both intra­molecular carb­oxy–carboxyl­ate O—H⋯O and imidazole–oxide N—H⋯O hydrogen bonds. Iin the crystal, cation–carboxyl­ate N—H⋯O and cation–imidazole N—H⋯N hydrogen bonds result in chains along the *b* axis.

## Related literature

For the structures of compounds with similar ligands, see: Chen (2008 ▶; Chen *et al.* (2011 ▶); Sun *et al.* (2005 ▶). For the synthesis of the ligand, see: Sun *et al.* (2006 ▶). 
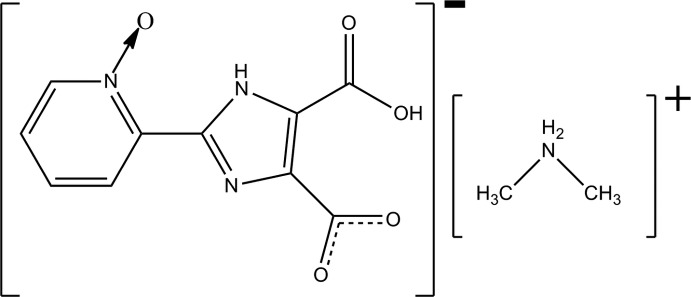


## Experimental

### 

#### Crystal data

C_2_H_8_N^+^·C_10_H_6_N_3_O_5_^−^
*M* *_r_* = 294.27
Monoclinic, 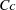

*a* = 10.9690 (18) Å
*b* = 17.305 (3) Å
*c* = 8.0160 (13) Å
β = 120.901 (2)°
*V* = 1305.6 (4) Å^3^
*Z* = 4
Mo *K*α radiation
μ = 0.12 mm^−1^
*T* = 298 K
0.32 × 0.28 × 0.26 mm

#### Data collection

Bruker APEXII area-detector diffractometer
Absorption correction: multi-scan (*SADABS*; Sheldrick, 1996 ▶) *T*_min_ = 0.963, *T*_max_ = 0.970
3782 measured reflections
1419 independent reflections
1204 reflections with *I* > 2σ(*I*)
*R*_int_ = 0.025

#### Refinement

*R*[*F* ^2^ > 2σ(*F* ^2^)] = 0.036
*wR*(*F* ^2^) = 0.085
*S* = 1.05
1419 reflections
193 parameters
2 restraints
H-atom parameters constrained
Δρ_max_ = 0.14 e Å^−3^
Δρ_min_ = −0.19 e Å^−3^

### 

Data collection: *APEX2* (Bruker, 2004 ▶); cell refinement: *SAINT* (Bruker, 2004 ▶); data reduction: *SAINT*; program(s) used to solve structure: *SHELXS97* (Sheldrick, 2008 ▶); program(s) used to refine structure: *SHELXL97* (Sheldrick, 2008 ▶); molecular graphics: *SHELXTL* (Sheldrick, 2008 ▶); software used to prepare material for publication: *SHELXTL*.

## Supplementary Material

Crystal structure: contains datablock(s) I, global. DOI: 10.1107/S1600536812033557/zs2224sup1.cif

Structure factors: contains datablock(s) I. DOI: 10.1107/S1600536812033557/zs2224Isup2.hkl

Supplementary material file. DOI: 10.1107/S1600536812033557/zs2224Isup3.mol

Supplementary material file. DOI: 10.1107/S1600536812033557/zs2224Isup4.cml

Additional supplementary materials:  crystallographic information; 3D view; checkCIF report

## Figures and Tables

**Table 1:** Hydrogen-bond geometry (Å, °)

*D*—H⋯*A*	*D*—H	H⋯*A*	*D*⋯*A*	*D*—H⋯*A*
N4—H4*B*⋯N1^i^	0.90	2.49	3.166 (3)	132
N4—H4*B*⋯O1^i^	0.90	2.11	2.933 (3)	151
N4—H4*A*⋯O1^ii^	0.90	1.95	2.806 (3)	159
O3—H3⋯O2	0.82	1.64	2.455 (3)	170
N2—H2⋯O5	0.86	2.06	2.603 (3)	120

Symmetry codes: (i) 
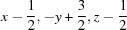
; (ii) 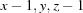.

**Fig. 1.**
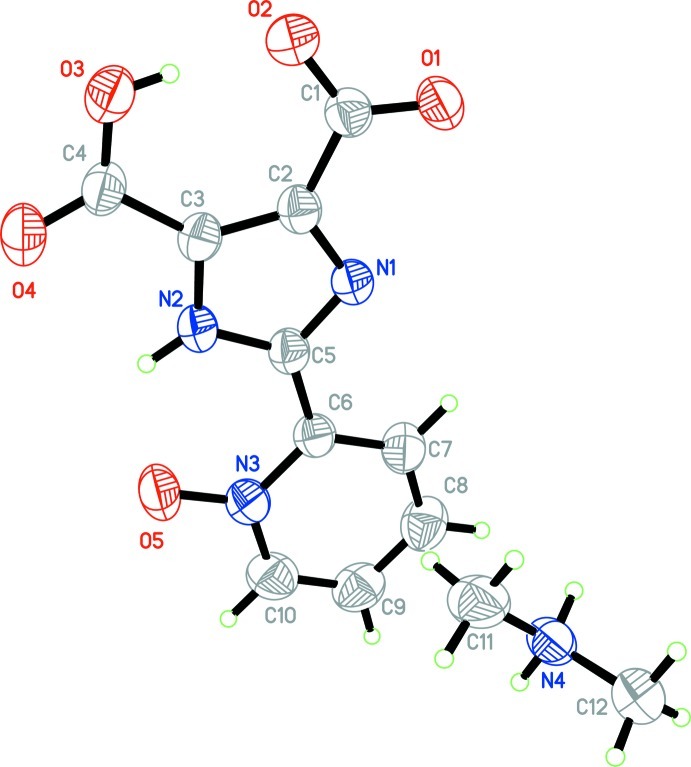
Molecular conformation and atom-numbering scheme for the title compound, with displacement ellipsoids drawn at the 50% probability level.

## supplementary crystallographic information

### Comment

Imidazole-4,5-dicarboxylic acid and its derivatives have a variety of coordination modes as ligands in the formation of metal complexes (Chen, 2008; Sun *et al.*, 2005), which include those with the lanthanide metals (Chen *et al.*, 2011). In the title salt, C_2_ H_8_N^+^ C_10_H_6_N_3_O_5_^-^, (Fig. 1), which consists of a dimethylammonium cation and a 5-carboxy-2-(2-pyridyl-*N*-oxide)-1*H*-imidazole-4-carboxylate anion, the anion is essentially planar [maximum deviation from the l.s. plane = 0.046 (5) Å], with the dihedral angle between the rings of 2.7 (2)Å. This conformation is maintained by the presence of both intramolecular carboxyl O—H···O and imidazole N—H···O_oxide_ hydrogen bonds while intermolecular cation N—H···O_carboxyl_ and N—H···N_imidazole_ hydrogen bonds (Table 1) give a one-dimensional chain structure.

### Experimental

The ligand,(4,5-dicarboxy-1*H*-imidazol-2-yl)pyridine-1-oxide was prepared by the method reported in the literature (Sun *et al.*, 2006). A diluted dimethylamine aqueous solution was added dropwise to an ethanolic solution of the ligand until the pH reached 7.4. Crystals of the title compound suitable for X-ray analysis were obtained after a few days of slow evaporation of the solvent.

### Refinement

Hydrogen atoms were placed at calculated positions (C—H = 0.95–0.99 Å, N—H = 0.90 Å and O—H = 0.82 Å) and were treated as riding on their parent atoms, with *U*_iso_(H) = 1.2–1.5*U*_eq_(C, N, O). In the absence of a suitable heavy atom, Friedel pairs were averaged in the refinement.

### Crystal data

**Table:**

C_2_H_8_N^+^·C_10_H_6_N_3_O_5_^−^	*F*(000) = 616
*M**_r_* = 294.27	*D*_x_ = 1.497 Mg m^−^^3^
Monoclinic, *C**c*	Mo *K*α radiation, λ = 0.71073 Å
Hall symbol: C -2yc	Cell parameters from 3600 reflections
*a* = 10.9690 (18) Å	θ = 1.3–28.0°
*b* = 17.305 (3) Å	µ = 0.12 mm^−^^1^
*c* = 8.0160 (13) Å	*T* = 298 K
β = 120.901 (2)°	Block, colourless
*V* = 1305.6 (4) Å^3^	0.32 × 0.28 × 0.26 mm
*Z* = 4	

### Data collection

**Table:**

Bruker APEXII area-detector diffractometer	1419 independent reflections
Radiation source: fine-focus sealed tube	1204 reflections with *I* > 2σ(*I*)
Graphite monochromator	*R*_int_ = 0.025
φ and ω scans	θ_max_ = 27.0°, θ_min_ = 2.4°
Absorption correction: multi-scan (*SADABS*; Sheldrick, 1996)	*h* = −14→12
*T*_min_ = 0.963, *T*_max_ = 0.970	*k* = −19→21
3782 measured reflections	*l* = −8→10

### Refinement

**Table:**

Refinement on *F*^2^	Primary atom site location: structure-invariant direct methods
Least-squares matrix: full	Secondary atom site location: difference Fourier map
*R*[*F*^2^ > 2σ(*F*^2^)] = 0.036	Hydrogen site location: inferred from neighbouring sites
*wR*(*F*^2^) = 0.085	H-atom parameters constrained
*S* = 1.05	*w* = 1/[σ^2^(*F*_o_^2^) + (0.0423*P*)^2^*P*] where *P* = (*F*_o_^2^ + 2*F*_c_^2^)/3
1419 reflections	(Δ/σ)_max_ < 0.001
193 parameters	Δρ_max_ = 0.14 e Å^−^^3^
2 restraints	Δρ_min_ = −0.19 e Å^−^^3^

### Special details

**Table:**

Geometry. All e.s.d.'s (except the e.s.d. in the dihedral angle between two l.s. planes) are estimated using the full covariance matrix. The cell e.s.d.'s are taken into account individually in the estimation of e.s.d.'s in distances, angles and torsion angles; correlations between e.s.d.'s in cell parameters are only used when they are defined by crystal symmetry. An approximate (isotropic) treatment of cell e.s.d.'s is used for estimating e.s.d.'s involving l.s. planes.
Refinement. Refinement of *F*^2^ against ALL reflections. The weighted *R*-factor *wR* and goodness of fit *S* are based on *F*^2^, conventional *R*-factors *R* are based on *F*, with *F* set to zero for negative *F*^2^. The threshold expression of *F*^2^ > σ(*F*^2^) is used only for calculating *R*-factors(gt) *etc*. and is not relevant to the choice of reflections for refinement. *R*-factors based on *F*^2^ are statistically about twice as large as those based on *F*, and *R*- factors based on ALL data will be even larger.

### Fractional atomic coordinates and isotropic or equivalent isotropic displacement parameters (Å^2^)

**Table:**

	*x*	*y*	*z*	*U*_iso_*/*U*_eq_	
C1	0.8048 (3)	0.88560 (14)	0.6235 (4)	0.0435 (6)	
C2	0.6875 (3)	0.92371 (14)	0.4485 (4)	0.0399 (6)	
C3	0.6630 (3)	1.00178 (15)	0.4067 (4)	0.0405 (6)	
C4	0.7352 (3)	1.07430 (16)	0.5114 (4)	0.0462 (7)	
C5	0.4999 (3)	0.93154 (13)	0.1662 (4)	0.0392 (6)	
C6	0.3776 (3)	0.90945 (15)	−0.0207 (4)	0.0408 (6)	
C7	0.3453 (3)	0.83297 (16)	−0.0743 (4)	0.0504 (7)	
H7	0.4041	0.7945	0.0095	0.060*	
C8	0.2281 (4)	0.81248 (17)	−0.2486 (5)	0.0581 (8)	
H8	0.2074	0.7607	−0.2825	0.070*	
C9	0.1414 (3)	0.86974 (19)	−0.3729 (4)	0.0578 (8)	
H9	0.0610	0.8570	−0.4910	0.069*	
C10	0.1752 (4)	0.94533 (19)	−0.3202 (5)	0.0584 (8)	
H10	0.1173	0.9838	−0.4047	0.070*	
C11	0.1058 (4)	0.83037 (18)	0.1270 (5)	0.0663 (9)	
H11A	0.0357	0.8668	0.1150	0.099*	
H11B	0.1698	0.8554	0.0960	0.099*	
H11C	0.1580	0.8113	0.2580	0.099*	
C12	−0.0512 (4)	0.71875 (19)	0.0443 (6)	0.0688 (9)	
H12A	0.0075	0.6984	0.1731	0.103*	
H12B	−0.0929	0.6769	−0.0464	0.103*	
H12C	−0.1250	0.7501	0.0399	0.103*	
N1	0.5853 (3)	0.88073 (11)	0.2989 (4)	0.0414 (5)	
N2	0.5433 (2)	1.00487 (12)	0.2268 (3)	0.0418 (5)	
H2	0.5024	1.0462	0.1629	0.050*	
N3	0.2913 (2)	0.96589 (13)	−0.1477 (3)	0.0468 (6)	
N4	0.0354 (3)	0.76580 (12)	−0.0071 (4)	0.0483 (5)	
H4A	−0.0202	0.7846	−0.1280	0.058*	
H4B	0.1019	0.7356	−0.0080	0.058*	
O1	0.8070 (2)	0.81390 (9)	0.6329 (3)	0.0522 (5)	
O2	0.8994 (2)	0.92884 (11)	0.7573 (3)	0.0584 (6)	
O3	0.8499 (2)	1.06669 (12)	0.6792 (3)	0.0583 (6)	
H3	0.8722	1.0209	0.6990	0.087*	
O4	0.6859 (3)	1.13664 (11)	0.4385 (4)	0.0669 (6)	
O5	0.3198 (3)	1.03909 (11)	−0.1066 (3)	0.0666 (7)	

### Atomic displacement parameters (Å^2^)

**Table:**

	*U*^11^	*U*^22^	*U*^33^	*U*^12^	*U*^13^	*U*^23^
C1	0.0445 (15)	0.0422 (14)	0.0441 (15)	0.0006 (13)	0.0229 (13)	0.0027 (13)
C2	0.0444 (15)	0.0369 (13)	0.0429 (15)	0.0007 (13)	0.0256 (13)	0.0014 (13)
C3	0.0437 (16)	0.0379 (13)	0.0433 (16)	−0.0040 (11)	0.0249 (14)	−0.0003 (11)
C4	0.0520 (19)	0.0372 (16)	0.0480 (18)	−0.0081 (12)	0.0248 (16)	−0.0012 (12)
C5	0.0427 (16)	0.0351 (14)	0.0415 (16)	−0.0004 (11)	0.0227 (14)	0.0032 (12)
C6	0.0405 (15)	0.0405 (13)	0.0404 (15)	−0.0010 (12)	0.0199 (13)	0.0046 (12)
C7	0.0578 (19)	0.0426 (14)	0.0482 (18)	−0.0033 (13)	0.0253 (17)	0.0002 (13)
C8	0.067 (2)	0.0522 (18)	0.0520 (18)	−0.0110 (16)	0.0279 (17)	−0.0071 (15)
C9	0.053 (2)	0.067 (2)	0.0443 (18)	−0.0098 (16)	0.0188 (16)	−0.0052 (15)
C10	0.0516 (19)	0.065 (2)	0.0466 (17)	0.0030 (16)	0.0167 (15)	0.0110 (15)
C11	0.0513 (19)	0.0587 (19)	0.067 (2)	0.0029 (15)	0.0145 (17)	−0.0142 (17)
C12	0.057 (2)	0.059 (2)	0.078 (2)	0.0050 (15)	0.0259 (19)	0.0192 (17)
N1	0.0419 (12)	0.0355 (11)	0.0410 (12)	−0.0004 (9)	0.0170 (10)	0.0029 (10)
N2	0.0450 (13)	0.0328 (11)	0.0447 (13)	0.0004 (9)	0.0211 (11)	0.0060 (9)
N3	0.0454 (14)	0.0442 (13)	0.0453 (14)	0.0005 (11)	0.0194 (13)	0.0065 (11)
N4	0.0450 (13)	0.0410 (12)	0.0476 (12)	0.0066 (10)	0.0157 (11)	0.0004 (11)
O1	0.0579 (12)	0.0383 (10)	0.0505 (12)	0.0056 (10)	0.0208 (11)	0.0057 (9)
O2	0.0515 (14)	0.0500 (13)	0.0542 (14)	−0.0042 (10)	0.0131 (12)	0.0017 (10)
O3	0.0610 (14)	0.0452 (12)	0.0562 (13)	−0.0110 (10)	0.0212 (12)	−0.0028 (10)
O4	0.0746 (16)	0.0372 (10)	0.0702 (15)	−0.0051 (12)	0.0238 (13)	0.0021 (11)
O5	0.0696 (15)	0.0361 (11)	0.0670 (15)	0.0016 (10)	0.0157 (13)	0.0075 (10)

### Geometric parameters (Å, º)

**Table:**

C1—O1	1.243 (3)	C9—C10	1.366 (4)
C1—O2	1.282 (3)	C9—H9	0.9300
C1—C2	1.485 (4)	C10—N3	1.361 (4)
C2—N1	1.367 (4)	C10—H10	0.9300
C2—C3	1.385 (4)	C11—N4	1.465 (4)
C3—N2	1.364 (3)	C11—H11A	0.9600
C3—C4	1.491 (4)	C11—H11B	0.9600
C4—O4	1.214 (3)	C11—H11C	0.9600
C4—O3	1.293 (4)	C12—N4	1.462 (4)
C5—N1	1.326 (3)	C12—H12A	0.9600
C5—N2	1.355 (3)	C12—H12B	0.9600
C5—C6	1.458 (4)	C12—H12C	0.9600
C6—N3	1.377 (3)	N2—H2	0.8600
C6—C7	1.380 (4)	N3—O5	1.306 (3)
C7—C8	1.373 (5)	N4—H4A	0.9000
C7—H7	0.9300	N4—H4B	0.9000
C8—C9	1.380 (4)	O3—H3	0.8200
C8—H8	0.9300		
			
O1—C1—O2	123.4 (3)	N3—C10—H10	119.1
O1—C1—C2	118.8 (3)	C9—C10—H10	119.1
O2—C1—C2	117.9 (2)	N4—C11—H11A	109.5
N1—C2—C3	110.4 (3)	N4—C11—H11B	109.5
N1—C2—C1	120.7 (2)	H11A—C11—H11B	109.5
C3—C2—C1	128.9 (3)	N4—C11—H11C	109.5
N2—C3—C2	104.8 (2)	H11A—C11—H11C	109.5
N2—C3—C4	120.4 (2)	H11B—C11—H11C	109.5
C2—C3—C4	134.7 (3)	N4—C12—H12A	109.5
O4—C4—O3	123.1 (3)	N4—C12—H12B	109.5
O4—C4—C3	120.0 (3)	H12A—C12—H12B	109.5
O3—C4—C3	116.8 (3)	N4—C12—H12C	109.5
N1—C5—N2	111.1 (2)	H12A—C12—H12C	109.5
N1—C5—C6	123.3 (2)	H12B—C12—H12C	109.5
N2—C5—C6	125.6 (2)	C5—N1—C2	105.44 (19)
N3—C6—C7	118.8 (3)	C5—N2—C3	108.2 (2)
N3—C6—C5	119.6 (2)	C5—N2—H2	125.9
C7—C6—C5	121.6 (3)	C3—N2—H2	125.9
C8—C7—C6	121.4 (3)	O5—N3—C10	119.2 (2)
C8—C7—H7	119.3	O5—N3—C6	121.1 (2)
C6—C7—H7	119.3	C10—N3—C6	119.7 (2)
C7—C8—C9	119.1 (3)	C12—N4—C11	113.1 (3)
C7—C8—H8	120.4	C12—N4—H4A	109.0
C9—C8—H8	120.4	C11—N4—H4A	109.0
C10—C9—C8	119.2 (3)	C12—N4—H4B	109.0
C10—C9—H9	120.4	C11—N4—H4B	109.0
C8—C9—H9	120.4	H4A—N4—H4B	107.8
N3—C10—C9	121.9 (3)	C4—O3—H3	109.5
			
O1—C1—C2—N1	−1.3 (4)	C6—C7—C8—C9	0.3 (4)
O2—C1—C2—N1	178.4 (2)	C7—C8—C9—C10	0.7 (5)
O1—C1—C2—C3	179.8 (3)	C8—C9—C10—N3	−0.6 (5)
O2—C1—C2—C3	−0.6 (4)	N2—C5—N1—C2	−0.6 (3)
N1—C2—C3—N2	−0.3 (3)	C6—C5—N1—C2	178.4 (2)
C1—C2—C3—N2	178.8 (2)	C3—C2—N1—C5	0.6 (3)
N1—C2—C3—C4	179.4 (3)	C1—C2—N1—C5	−178.6 (2)
C1—C2—C3—C4	−1.5 (5)	N1—C5—N2—C3	0.5 (3)
N2—C3—C4—O4	1.6 (4)	C6—C5—N2—C3	−178.6 (2)
C2—C3—C4—O4	−178.0 (3)	C2—C3—N2—C5	−0.1 (2)
N2—C3—C4—O3	−178.4 (2)	C4—C3—N2—C5	−179.8 (2)
C2—C3—C4—O3	2.0 (4)	C9—C10—N3—O5	178.8 (3)
N1—C5—C6—N3	177.7 (2)	C9—C10—N3—C6	−0.4 (4)
N2—C5—C6—N3	−3.4 (4)	C7—C6—N3—O5	−177.8 (2)
N1—C5—C6—C7	−2.2 (4)	C5—C6—N3—O5	2.3 (3)
N2—C5—C6—C7	176.7 (3)	C7—C6—N3—C10	1.4 (4)
N3—C6—C7—C8	−1.4 (4)	C5—C6—N3—C10	−178.5 (2)
C5—C6—C7—C8	178.5 (3)		

### Hydrogen-bond geometry (Å, º)

**Table:**

*D*—H···*A*	*D*—H	H···*A*	*D*···*A*	*D*—H···*A*
N4—H4*B*···N1^i^	0.90	2.49	3.166 (3)	132
N4—H4*B*···O1^i^	0.90	2.11	2.933 (3)	151
N4—H4*A*···O1^ii^	0.90	1.95	2.806 (3)	159
O3—H3···O2	0.82	1.64	2.455 (3)	170
N2—H2···O5	0.86	2.06	2.603 (3)	120

Symmetry codes: (i) *x*−1/2, −*y*+3/2, *z*−1/2; (ii) *x*−1, *y*, *z*−1.
